# Robust hypoxia-selective regulation of a retinal pigment epithelium-specific adeno-associated virus vector

**Published:** 2008-03-07

**Authors:** Christopher J. Dougherty, George W. Smith, C. Kathleen Dorey, Howard M. Prentice, Keith A. Webster, Janet C. Blanks

**Affiliations:** 1 Charles E. Schmidt College of Biomedical Science, Florida Atlantic University, Boca Raton, FL; 2 Vascular Biology Institute, University of Miami Miller School of Medicine, Miami, FL; 3 Charles E. Schmidt College of Science, Center for Complex Systems and Brain Sciences, Florida Atlantic University, Boca Raton, FL

## Abstract

**Purpose:**

To develop an hypoxia-regulated retinal pigment epithelium (RPE)-specific adeno-associated virus (AAV) gene transfer platform that exploits hypoxia as a physiologic trigger for an early antiangiogenic treatment strategy directed at arresting neovascularization in the eye.

**Methods:**

Tissue-specific and hypoxia-regulated expression vectors were constructed with tandem combinations of hypoxia responsive elements and aerobically silenced elements (HRSE) that together induce gene expression in hypoxia and suppress it in normoxia. For RPE-specific expression, the HRSE and a (6x) HRE (hypoxia responsive element) oligomer were ligated upstream of the Rpe65 promoter in a pGL3 firefly luciferase plasmid (*pGL3-HRSE-6xHRE-Rpe65*). The cell specificity of this novel hybrid promoter was tested in human RPE (ARPE-19), human glioblastoma, rat C6 glioma, mouse hippocampal neurons, and human embryonic kidney cell lines. Expression of all cell types in normoxia, and following 40 h hypoxia, was analyzed by dual luciferase assay. After confirmation of its tissue-specificity and hypoxia-inducibility, the hybrid promoter construct was integrated into a replication-deficient AAV delivery system and tested for cell-selectivity and hypoxia-inducible green fluorescent protein (GFP) reporter expression.

**Results:**

The HRSE-6xHRE-Rpe65 promoter was highly selective for RPE cells, strongly induced in hypoxia, and similar in activation strength to the cytomegalovirus (CMV) promoter. The AAV.HRSE.6xHRE.Rpe65 vector induced robust GFP expression in hypoxic ARPE-19 cells, but elicited no GFP expression in hypoxia in other cell types or in normoxic ARPE-19 cells.

**Conclusions:**

The hypoxia-regulated, aerobically-silenced RPE-targeting vector forms a platform for focal autoregulated delivery of antiangiogenic agents in hypoxic regions of the RPE. Such autoinitiated therapy provides a means for early intervention in choroidal neovascularization, when it is most sensitive to inhibition.

## Introduction

Gene transfer of an antiangiogenic gene to areas of focal hypoxia in the retina or retinal pigment epithelium (RPE) may provide a useful approach for treatment of choroidal neovascularization (CNV) in the “wet” form of age-related macular degeneration (AMD) [1,2]. Hypoxia-induced changes in tissue physiology trigger ocular pathology [3-7]. This hypoxia-induced activation of gene expression can also serve as a physiologic switch for activation of a therapeutic response.

The sensing of tissue oxygen for such a switch occurs through changes in hypoxia-inducible factor-1 (HIF-1). The physiologic response to tissue hypoxia is initiated by the binding of the HIF-1 transcription factor to the hypoxia response element (HRE) [8,9]. HIF-1 is a basic helix–loop–helix protein formed by heterodimerization of HIF-1α and HIF-1β, which are both constitutively expressed [10,11]. Under normoxic conditions, oxygen-dependent hydroxylation of prolyl residues of HIF-1α targets it for rapid ubiquination and degradation by the proteosome; in contrast, HIF-1β is unregulated [12,13].

In hypoxic conditions, heterodimerization of HIF-1α and HIF-1β forms HIF-1, which translocates to the nucleus to bind HREs and upregulate gene expression [14]. The core consensus sequence for the HRE is (A/G)CGT(G/C)C, which occurs in the 5′ or 3′ flanking sequences of greater than 60 hypoxia-regulated genes identified to date [15]. Thus implementation of HRE consensus sequences exploits an endogenous, exquisitely sensitive regulatory pathway as a physiologic switch for hypoxia-regulated delivery of a therapeutic gene.

A major potential concern regarding gene therapy is that surrounding tissue may be adversely affected after prolonged exposure to the transgene. Most existing gene therapy vectors express the transferred gene product at a constant level in all tissue types exposed to the vector, resulting in high level transgene expression outside of the intended therapeutic region. Adenoviral-mediated transgene expression typically persists only days to weeks before clearance by the immune system and often stimulates an inflammatory response [16]. Because of rapid clearance chronic expression has not been a major concern. More recently, novel adeno-associated virus (AAV) vectors have been designed to stay functional for years and possibly the lifetime of the patient; therefore extended exposure to elevated transgene levels in adjacent tissue might produce a higher risk for dysplasia.

Hypoxia is found in several pathological conditions and has been recognized as a potential pathological regulator for gene therapy. Hypoxia-regulated gene therapy has been successfully demonstrated in several disease models, including cardiac ischemia [17,18], cancer [19-22], and ocular angiogenesis [23]. Tissue-specific vectors have already been constructed for selective gene expression in neurons [24], endothelial cells [25], cardiac tissue [18,26], photoreceptors [27,28], and RPE [29]. The RPE is an excellent target for gene therapy in the posterior eye due to its location between the retina and the choriocapillaris, and adjacent to Bruch’s membrane where inflammatory processes contribute to AMD. It may therefore be appropriate to target the RPE in either geographic atrophy or CNV.

Therapeutic targeting of the RPE can be achieved by using the Rpe65 promoter, which is constitutively activated in the RPE but not in other retinal cell types [30]. Rod and cone photoreceptor function in a canine model of Leber congenital amaurosis was successfully restored with a cell-specific therapy targeted to the RPE using the Rpe65 promoter [31]. Coupling this promoter with a hypoxia-regulated cassette would create a gene therapy that would support delivery in focal hypoxia. An added benefit is that gene therapy could theoretically be titrated to the degree of hypoxia since more severe hypoxia would result in greater expression of the transgene. The optimal inducible expression system for delivery of an antiangiogenic agent should have low basal promoter activity in the nontherapeutic or normoxic environment. After gene transfer, the therapy should remain quiescent until activated by a pathological trigger such as hypoxia. Data presented here documents hypoxia-regulated expression of green fluorescent protein (GFP) within RPE cells, but not other cell lines, transfected with AAV-vectors. Thus, although other cell types may be transfected with the vector, transgene expression will be restricted to foci of hypoxic RPE cells.

## Methods

### Cell cultures and reagents

The ARPE-19 (human retinal pigment epithelium), HepG2 (human hepatoma), and A-172 (human glioblastoma) cell lines were purchased from the American Type Culture Collection (Manassas, VA). HEK-293 (human embryonic kidney) cells were purchased from Stratagene (La Jolla, CA). HT22 (mouse hippocampal neurons) cells were a kind gift from Dr. David R. Schubert (The Salk Institute, San Diego, CA). C6 glioma cells were a kind gift from Dr. Ajay Verma (Uniformed Health Services University, Bethesda, MD). Cell cultures were maintained in Dulbecco’s modified eagle medium (DMEM) (Cellgro-Mediatech, Herndon, VA) supplemented with 10% fetal bovine serum (HyClone, Logan, UT) and 1% penicillin/streptomycin (Cellgro-Mediatech).

**Figure 1:**
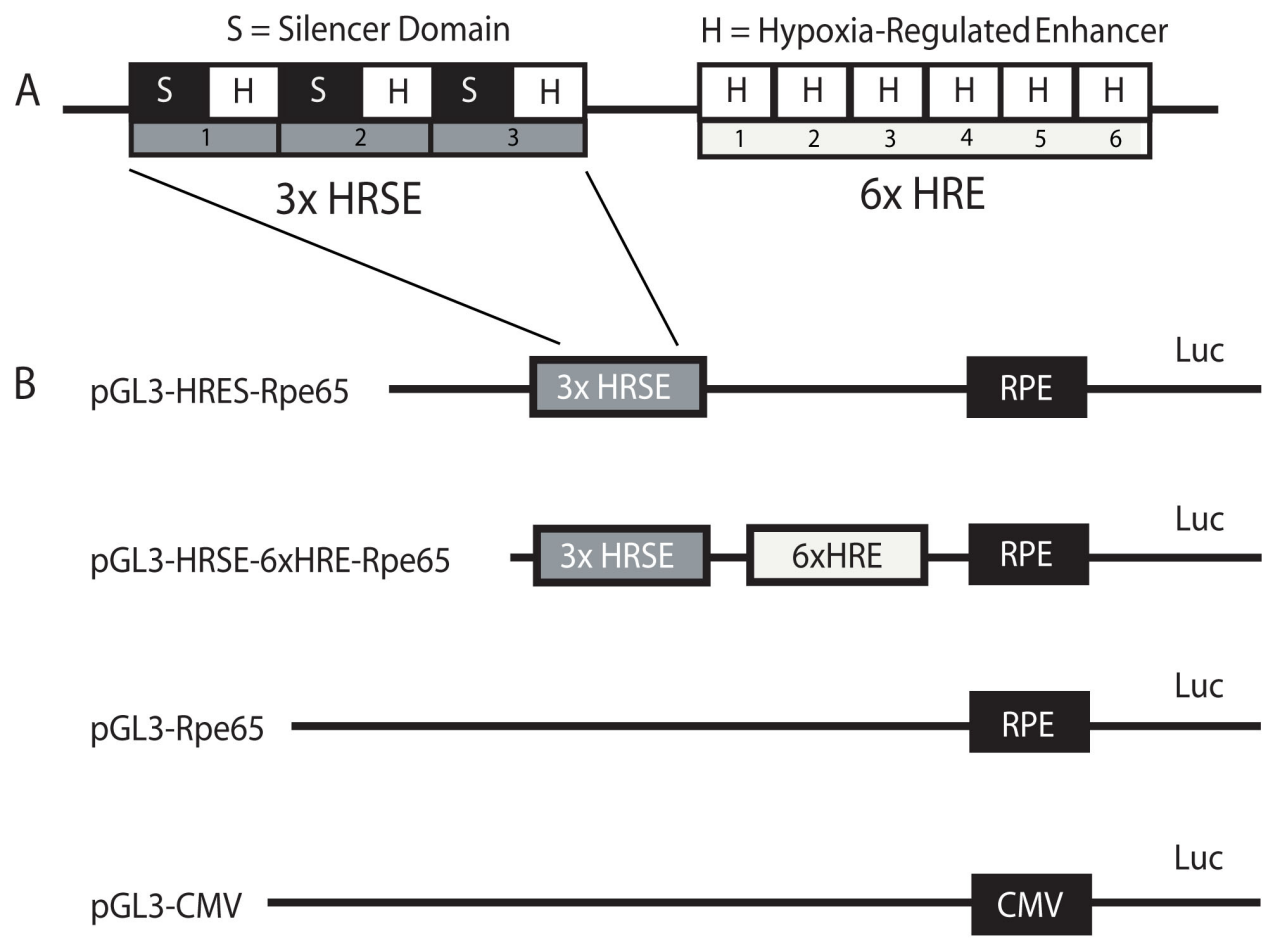
Diagram of the aerobically silenced, hypoxia-regulated promoter enhancer. **A:** Three copies of the neuron-restrictive silencer element (NRSE) and three copies of the hypoxia-regulated enhancer (HRE) were placed in alternating tandem order to form the hypoxia-regulated silenced element (HRSE), which is conditionally silenced during aerobic periods and robustly induced in hypoxia. This 3xHRSE domain is composed of the NRSE (*S*) and the HRH (*H*) tandemly repeated three times. This domain is ligated to a 6xHRE domain, which is six additional tandem repeats of the HRE. **B:** The table graphic represents the four pGL3-based firefly luciferase reporter plasmids constructed to test the relative activities of the hybrid and control promoters.

### Design and construction of retinal pigmented epithelium-specific and hypoxia-inducible promoters for reporter gene expression plasmids

The HRE sequence is from the human phosphoglycerate kinase (PGK) gene in the sense orientation 5′-TGT CAC GTC CTG CAC GAC GTA-3′. The neuron-restrictive silencer element is the sequence from the human synapsin I gene in the sense orientation, 5′-TTC AGC ACC GCG GAC AGT GCC-3′ [32]. The Rpe65 promoter (−325 to +52) was derived from the 5′ upstream sequence of the Rpe65 gene (a generous gift from Dr. Michael Redmond) [30]. Three copies of the neuron-restrictive silencer element and three copies of the HRE were placed in alternating tandem order to form the hypoxia-regulated silenced element (HRSE; kindly provided by Dr. Keith Webster), which is a promoter designed to be conditionally silenced in normoxia and robustly induced during in hypoxia (Figure 1A**)**. Regulation of gene expression by combined tandem arrays of HRE and silencer elements has been described previously [33,34]. Additionally, six tandem HRE copies were synthesized (e-oligos, Hawthorne, NY) into one oligomer (6xHRE), which functions to enhance the hypoxic activity of the promoter (K. Webster, personal communication). This oligomer was synthesized as two 144 bp synthetic complementary oligos annealed into a double-stranded oligonucleotide with *Sac* I restriction sites incorporated at each termini. The HRSE and 6xHRE regions were ligated upstream of the RPE65 promoter for expression analysis in a pGL3 luciferase reporter vector (Promega, Madison, WI), (Figure 1B). The reporter plasmids constructed were pGL3-HRSE, pGL3-HRSE-Rpe65 and pGL3-HRSE-6xHRE-Rpe65. To gauge the relative strength of the hypoxic induction, expression plasmids encoding individual Rpe65 or cytomegalovirus (CMV) promoters were also constructed for direct comparison of the hybrid promoter to the parent tissue specific promoter (Rpe65) and to CMV.

### Plasmid transfection, hypoxia treatment, and dual luciferase assay

The pGL3 based reporter constructs were transiently transfected by polycationic liposomes (GeneFector*™*, VennNova Inc., Pompano Beach, FL) with pGL3-TK-Renilla (transfection control plasmid) into ARPE-19, HEK-293, HT22 and C6 glioma cells cultured on 24 well plates (Greiner Bio-One, Monroe, NC) at a total concentration of 1µg DNA per well. The average transfection efficiency was around 70%. Cells were maintained in Dulbecco’s modified eagle medium (DMEM) supplemented with 10% fetal bovine serum and 1% penicillin/streptomycin and fed one hour before transfer to either the Bactron Anaerobic Environmental Chamber (Sheldon Manufacturing Inc., Cornelius, OR) maintained at pO_2_=1.0%, or a cell culture incubator (pO_2_=20.8%) for the experimental time course of 40 h at 37 °C. Chamber hypoxia was maintained at 1% O_2_, 89% N, 5% CO_2_, 5% H. Chamber oxygen was continuously monitored using a MI-BASO2 oxygen electrode (Microelectrodes Inc., Bedford, NH). Culture plates were introduced to and removed from the chamber through a small side bypass chamber. Cultures were fed with hypoxic media inside the chamber using an integrated glove system. Cells were isolated from either normoxia or hypoxia then lysed and assayed using the dual luciferase assay per the manufacturer's protocol (Dual Luciferase Assay Kit; Promega, Madison, WI). Results are presented as fold induction with respect to a normalized normoxic luciferase activity measured in relative light units (rlu). All experiments were repeated at least twice and measured in triplicate each time.

**Figure 2:**
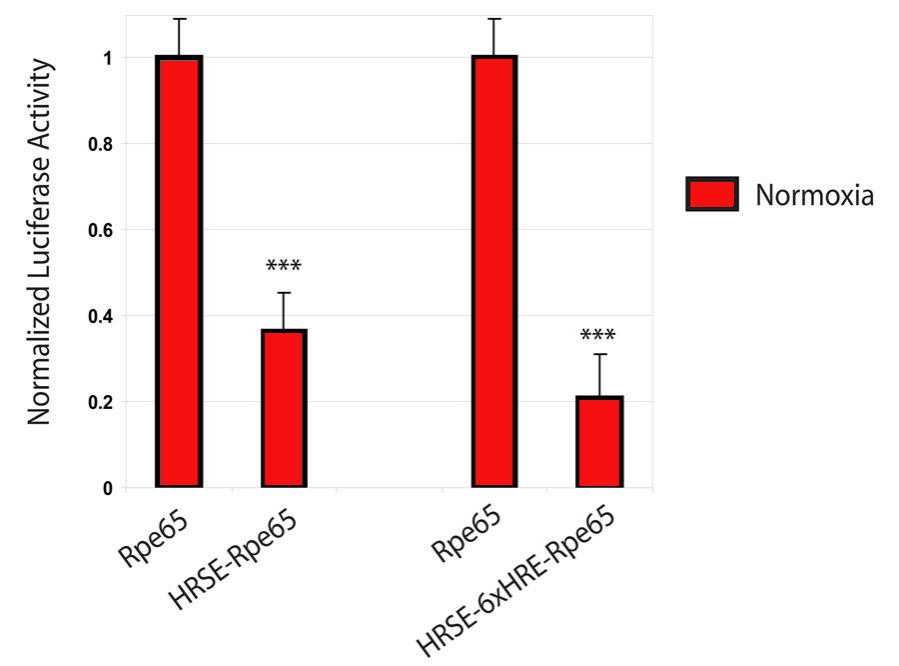
Normoxic dual luciferase expression using the Rpe65 promoter and the HRSE-6xHRE-Rpe65 promoter in transiently transfected ARPE-19 cells. ARPE-19 cells were transiently transfected by cationic lipid and exposed to forty hours of normoxia. The silencing of basal activity from the RPE65 promoter, under normal aerobic conditions, was due to the integration of the three neuron-restrictive silencer elements within the hypoxia-regulated aerobically silenced element (HRSE). The activity of all promoters tested was normalized to the Rpe65 promoter. The addition of the silencer elements in the HRSE reduced the normoxic activity of the Rpe65 promoter by 64% from 1.0 to 0.4±0.1 (n=6, p<0.001). Addition of six hypoxia-regulated elements (HREs) in HRSE-6xHRE-Rpe65 reduced the normoxic activity of the Rpe65 promoter by 79% from 1.0 to 0.2±0.1 (n=8, p<0.001). Asterisks (*) indicates statistical significance.

### Construction and production of recombinant adeno-associated virus vectors

The Helper-Free AAV System© (Stratagene) permits the production of replication deficient recombinant human AAV, serotype-2 without the use of a helper virus. The pAAV-IRES-hrGFP shuttle plasmid was subcloned to remove the CMV promoter using a *Mlul/Sall* restriction enzyme digest to allow the introduction of the HRSE-6xHRE-Rpe65 cassette. The HRSE-6xHRE-Rpe65 promoter cassette was cloned into the pAAV-IRES-hrGFP expression plasmid using the multiple cloning site. Propagation of the recombinant AAVs was conducted per the manufacturer’s protocol. Briefly, AAV-293 cells were triple transfected by the calcium phosphate method using the pAAV-HRSE-6xHRE-Rpe65 in conjunction with pAAV-RC and pHelper plasmids for 66 to 72 h before freeze thaw lysis and purification of AAV. Both experimental AAV.HRSE.6xHRE.Rpe65.GFP and control AAV.CMV.GFP viruses were constructed.

### Infection of cell culture with adeno-associated virus

ARPE-19, HT22, HEK-293, and C6 glioma cell cultures were transfected with AAV.HRSE.6xHRE.Rpe65.GFP at a moiety of infection of 10 and cultured for seven days before experimental exposure to normoxia or hypoxia for 40 h before fluorescence microscopy to detect the expression of GFP. On average, 99% of the cells were transfected with AAV.

**Figure 3:**
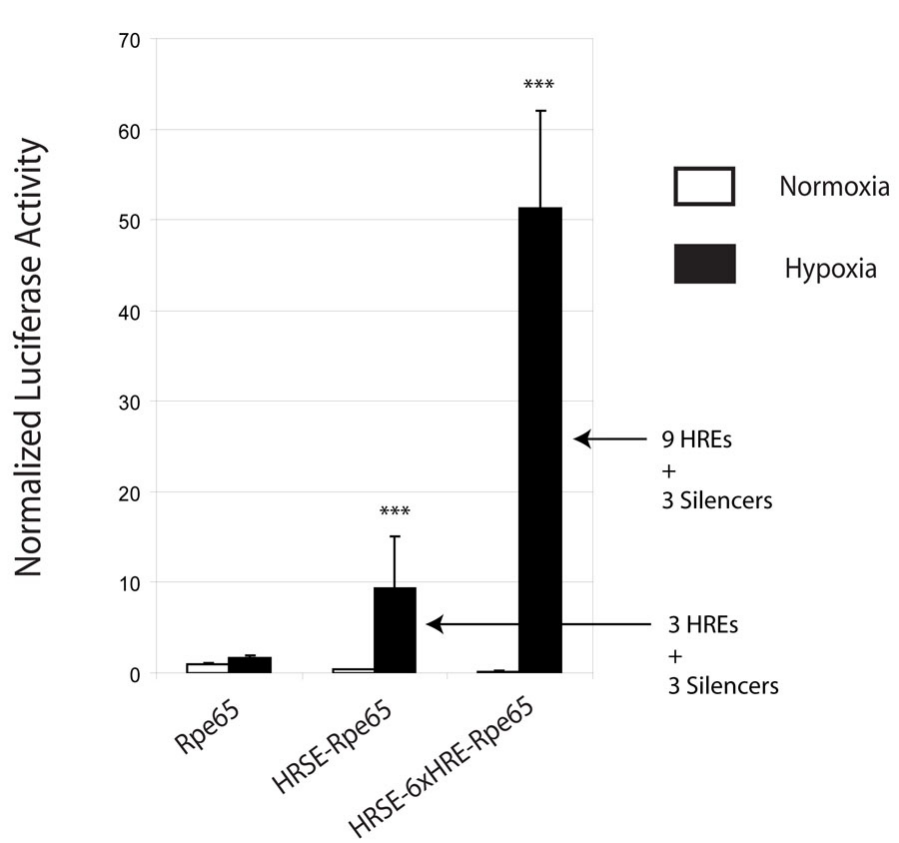
Hypoxic dual luciferase expression in ARPE-19 cells transfected with the Rpe65 promoter, the HRSE-Rpe65 promoter containing three hypoxia-regulated elements, and the HRSE-6xHRE-Rpe65 promoter. The addition of the three hypoxia-regulated elements (HREs) in HRSE-Rpe65 accounts for a modest increase in basal Rpe65 inducibility (black). Transiently transfected cell lines were exposed to forty hours of hypoxia (black) or normoxia (white). All promoter activity was normalized to the aerobic activity of the Rpe65 promoter. A small hypoxic induction of 1.6±0.3 (n=7, p>0.05) fold was identified in the native Rpe65 promoter. Ligation of an upstream HRSE to the Rpe65 promoter resulted in a 9.4±5.8 (n=9, p<0.001) fold hypoxic induction due to three HREs present in the HRSE. Addition of the 6xHRE oligomer brings the total number of HREs to nine and accounts for the 51.3±10.8 (n=10, p<0.001) fold increase in hypoxia inducibility over aerobic baseline. Asterisks (*) indicate statistical significance.

### Statistical analysis

Results are expressed as mean ± standard deviation. Differences between groups were evaluated by two-tailed Student’s t-test. The significance of the hypoxic induction and silencing measured by DLA was determined by ANOVA using InStat 3.0b statistical software for Apple OS X.

## Results

### Conditionally silenced gene expression in normoxia

The aerobic activity profile of the basal Rpe65 promoter was compared to the HRSE-6xHRE-Rpe65 promoter using a DLA. Transiently transfected ARPE-19 cells were exposed to 40 h of normoxia. The activity of the Rpe65 promoter was normalized to one. Interestingly, addition of the three silencer elements in the (3x) HRSE to the Rpe65 promoter reduced the normoxic activity of the Rpe65 promoter by 60% from 1.0 to 0.4±0.1 (n=6, p<0.001) (Figure 2). With the addition of six more HREs, the normoxic activity of the Rpe65 promoter was reduced by 80% from 1.0 to 0.2±0.1 (n=8, p<0.001).

**Figure 4:**
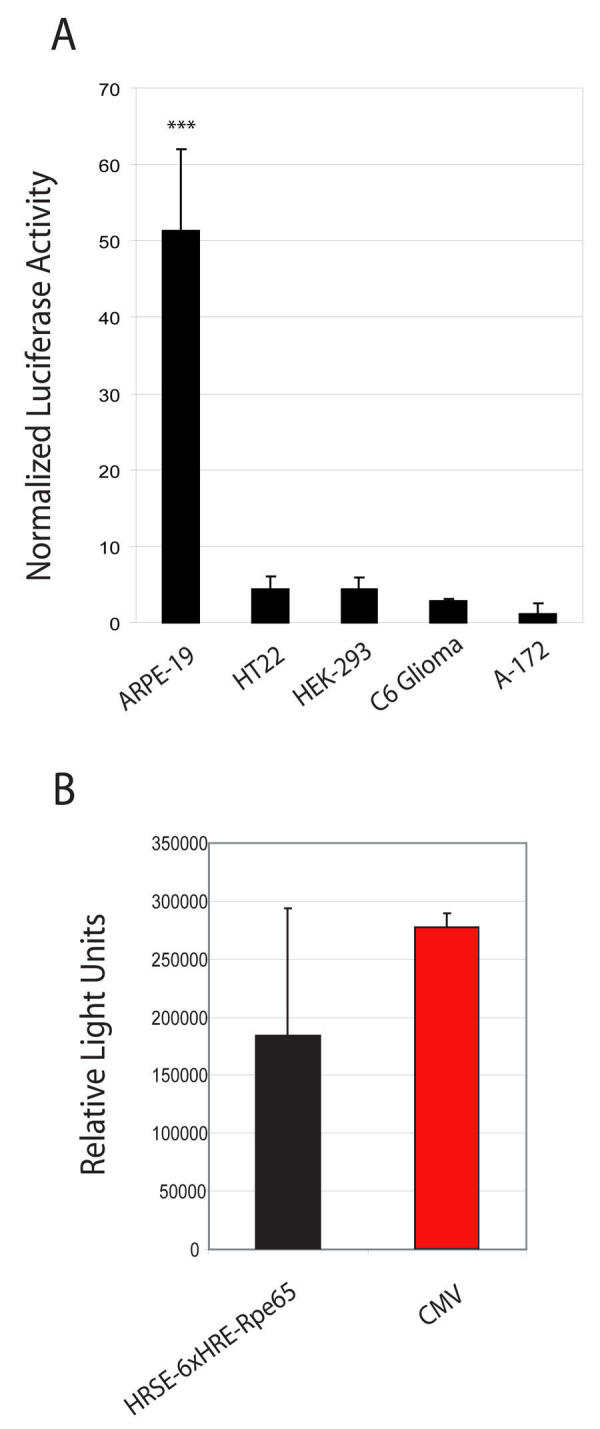
Demonstration of cell-specificity. **A:** Comparison of hypoxia induced dual luciferase activity of the HRSE-6xHRE-RPE65 promoter in retinal pigmented epithelium (ARPE-19) cells with other cell types. The activation of the HRSE-6xHRE-Rpe65 promoter in ARPE-19 cells exposed to 1% oxygen for 40 hours was 51.3±10.8 (n=10, p<0.001) fold over aerobic activity. The non-epithelial cell lines displayed a relatively minimal activation of the HRSE-6xHRE-Rpe65 promoter throughout the same hypoxic time course confirming a hypoxia-inducible and cell-specific gene expression regulated by the HRSE-6xHRE-Rpe65 promoter construct. Hypoxia-mediated promoter activation for the tested cell lines were: HT22 mouse hippocampal neuron (4.3±1.6, n=9, p<0.001), HEK-293 human embryonic kidney (3.3±2.5, n=6, p<0.001), C6 glioma (2.8±0.3, n=5, p<0.001) and A-172 glioblastoma (1.2±1.4, n=3, p<0.001). **B:** The relative strength of the HRSE-6xHRE-Rpe65 promoter in hypoxia is comparable to cytomegalovirus (CMV). The histogram illustrates that the HRSE-6xHRE-Rpe65 promoter activity was similar to the CMV promoter under hypoxic conditions. Under aerobic conditions the CMV promoter activity remains strong while the HRSE-6xHRE-Rpe65 promoter is conditionally silenced. Asterisks (*) indicate statistical significance.

### Hypoxia-inducible gene expression of HRSE-6xHRE-Rpe65

Progressive increases in the number of HREs led to a 51-fold increase in gene depression in hypoxia. Enhanced expression of each construct occurred in ARPE-19 cells transfected by cationic lipid and cultured for 40 h in hypoxia. Relative to the aerobic activity of the Rpe65 promoter, the basal Rpe65 promoter activity was increased to a small degree by 1.6±0.3 fold (n=7, p>0.05) in hypoxia (Figure 3). Ligation of this Rpe65 promoter to an upstream HRSE resulted in a 9.4±5.7 fold (n=9, p<0.001) induction. Hypoxic induction was further increased with the addition of six tandem HREs inserted at a position between the HRSE and the Rpe65 promoter. With a total number of nine HREs, the HRSE-6xHRE-Rpe65 promoter in hypoxia increased 51.2±10.8 fold (n=10, p<0.001; see Figure 3). Preliminary data indicate that 40 h of hypoxia is sufficient to induce a maximal response of this hypoxia-regulated promoter (data not shown).

### Inducible gene expression of HRSE-6xHRE-Rpe65 in non-target cell lines

To further characterize cell-specificity of the HRSE-6xHRE-Rpe65 promoter construct, we transiently transfected several additional nontarget cell lines, including HT22, HEK-293, C6 Glioma, HepG2, and A-172, exposing them to hypoxia for 40 h before analysis by DLA. In contrast to the 51.2 fold increase in luciferase activity observed in hypoxic ARPE-19 cells, the promoter supported only minimal (more than tenfold smaller) response to hypoxia in the nontarget cell lines (Figure 4A). Hypoxia-mediated promoter activation in these tested cell lines was HT22 (4.3±1.6; n=9, p<0.001), HEK-293 (3.3±2.5; n=6, p<0.001), C6 glioma (2.8±0.3; n=5, p<0.001), and A-172 (1.2±1.4; n=3, p<0.001).

To predict the level of transgene expression in hypoxic ARPE-19 cells, we used a DLA to compare the promoter strengths of the HRSE-6xHRE-Rpe65 promoter construct and a CMV promoter construct. Figure 4B indicates that the HRSE-6xHRE-Rpe65 promoter activity was almost as strong as the CMV promoter under hypoxic conditions. Under aerobic conditions, the CMV promoter activity remained strong whereas the HRSE-6xHRE-Rpe65 promoter was conditionally silenced.

### Cell specificity of inducible green fluorescent protein expression in AAV-HRSE-6xHRE-Rpe65 infected cell lines

**Figure 5:**
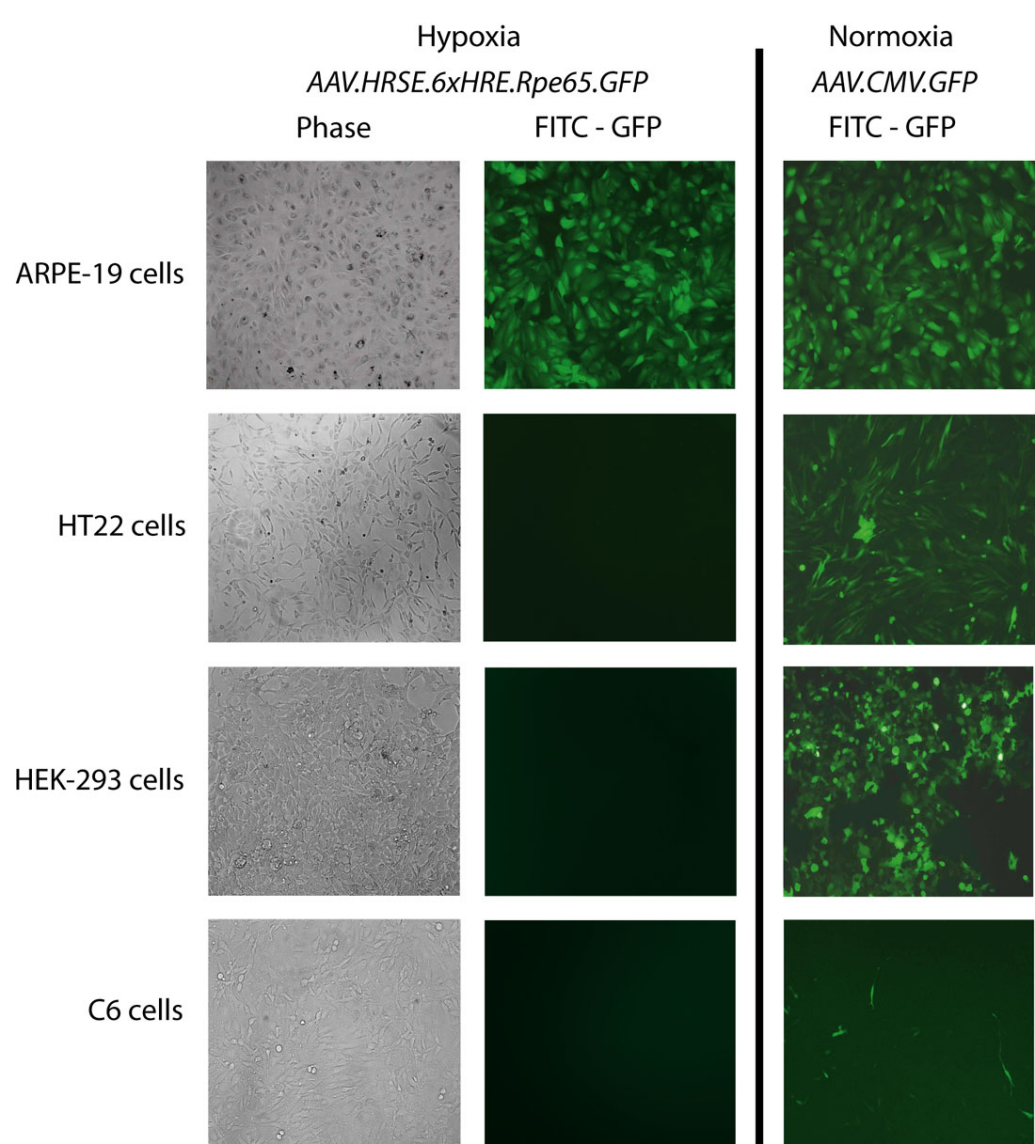
Hypoxia-inducible green fluorescent expression is specific to ARPE-19 cells transfected with AAV.HRSE.6xHRE.Rpe65.GFP. Hypoxia-inducible green fluorescent protein (GFP) expression is specific to RPE (ARPE-19) cells transfected with the AAV.HRSE.6xHRE.Rpe65.GFP vector. Cells were transfected with AAV.HRSE.6xHRE.Rpe65.GFP and cultured for seven days before forty hours of hypoxia (pO_2_=1%). Hypoxia-inducible GFP expression is specific to only ARPE-19 cells and was not detected in transfected HT22 mouse hippocampal neurons, HEK-293 human embryonic kidney or C6 glioma cell lines cultured under similar conditions. Duplicate phase contrast images detail the morphology of the transfected cell lines in column one. In column three, representative images of control cultures infected with AAV.CMV.GFP express GFP constitutively during normoxic conditions.

The HRSE-6xHRE-Rpe65 promoter was used to produce a replication-deficient AAV serotype 2 for cell culture transfection. Figure 5 shows representative phase and fluorescence images of the different cell lines after 40 h of hypoxia. Hypoxic ARPE-19 cells clearly demonstrated strong induction of GFP. The other cell lines did not produce detectable GFP under these conditions. Constitutive expression of GFP in normoxic cells transfected with AAV.CMV.GFP is also shown in Figure 5**.** When the transfected ARPE-19 cells were returned to normoxia, the GFP level declined rapidly and was barely detectable at 72 h (data not shown). The fact that GFP has a half-life of 26 h suggests that the promoter is rapidly suppressed in oxygen.

## Discussion

This report demonstrates a new hypoxia-regulated gene expression platform specific for the RPE. Induction of hypoxia-responsive promoters in disease models in vivo has previously been reported [23,26,35]. To achieve hypoxia-regulated gene expression, previous reports have employed different HREs, such as from PGK [36]. Specifically, Boast et al. achieved more than a 50-fold hypoxic induction with PGK-HREs. Bainbridge et al. later constructed a hypoxia-responsive vector by placing three PGK-HREs upstream of a GFP reporter in a recombinant AAV [23]. The vector drove retinal GFP expression in a mouse model of laser-induced retinal hypoxia. One drawback of this vector was a readily detectable expression of GFP in control mice, indicating significant basal activity of the HRE enhancer/promoter under aerobic conditions. Our approach differs in that the basal activity of our HREs is tempered by engineered silencer elements that reduce the basal transcriptional activity of the HREs and the downstream tissue-specific promoter.

Potentially deleterious effects of gene therapy can be minimized by limiting expression of a transgene to the area of diseased tissue, so that therapy occurs only at the right time, and in the right place. Expression of a transgene at the “wrong” time or place (i.e., in healthy tissue) might result in detrimental consequences for tissue survival. Our cell-specific, hypoxia-regulated platform would deliver antiangiogenic agents only in the immediate locale of hypoxic RPE cells.

Vectors containing only multiple copies of HREs are not optimal because they exhibit high basal levels of expression in normoxia. To achieve maximal inducibility in response to hypoxia it is necessary to obtain very low levels of basal expression using a tissue-specific silencer that binds and blocks the promoter during aerobic exposure [37,38]. It has previously been demonstrated in vivo that a promoter containing a neuronal silencer element is inactive at normal oxygen in all tissues except brain, as its function is to silence neuronal gene expression in nonneuronal tissue [38]. Specifically, a promoter containing both copies of the neuronal silencer plus the HRE was induced several hundredfold by hypoxia in cardiac tissue with the use of a tissue-specific basal promoter [37,38]. These HRE enhancer elements were also constructed with multiple alternating copies of HREs and silencer elements. Furthermore cell culture and in vivo studies demonstrated that the hypoxia-regulated promoters have the potential to be robustly induced when employed in combination with different tissue-specific basal promoters [39,40].

With the incorporation of three HREs in the HRSE to the promoter, the hypoxia response increased from 1.6 fold with the basal Rpe65 promoter to 9.4 fold (Figure 3). In this novel paradigm, transcription is activated by hypoxia, when HIF-1 displaces the silencer protein and permits transcription. The use of these multiple neuronal-restrictive silencer elements (Figure 1) generated an impressive 80% reduction of basal Rpe65 promoter activity (Figure 2). This reflects the type of response expected from the silenced promoter in a normal oxygenated environment where expression of a transgene is not expected or required. The ability to titrate the amount of growth factor delivered to focal regions in the posterior eye would avoid “overtreating” a larger area where therapy is not required. This strict promoter regulation may prove useful in future gene therapy applications in the eye as well as in other organs.

Under normal aerobic conditions, the embedded silencer elements would function to diminish the “leakiness” typically observed in promoter constructs. The combination of aerobic silencing and strong hypoxic induction demonstrates that the promoter construct HRSE-6xHRE-Rpe65 is ideal for regulating hypoxic expression in ARPE-19 cells.

Although numerous reports document that ocular gene therapy mediated through viral vectors has the potential to treat neovascularization [41-46], to delay photoreceptor degeneration [47-50], and to suppress immune response [51], little attention has been paid to cell targets, the ocular location for optimal delivery of the therapeutic factor, or to the physiologic conditions in the retina being treated. Tight transcriptional regulation has recently been combined with cell-specific promoters, such as Rpe65 for RPE cells [30], to regulate both the level and location of transcription [52]. The cell-specific and hypoxia-regulated vector reported here is likely to lay the foundation for further rational design strategies for ocular gene therapy vectors.

Initially, it was predicted that the minimal activation of HRSE-6xHRE-Rpe65 quantitated in the non-RPE luciferase assays (Figure 4) would directly correlate with GFP expression in cells transfected with HRE-regulated AAVs. Transfected ARPE-19 cells exposed to hypoxic conditions did indeed produce a robust cytosolic GFP expression analogous to that seen in the luciferase assays, but there was no detectable GFP expression in the hypoxic non-target cell lines (Figure 5). These results are not surprising since luciferase assays are far more sensitive than detection of GFP by fluorescent microscopy. These sensitive changes in GFP production also may be detectable at the RNA level using RT–PCR.

The role of hypoxia in the pathophysiology of vascular endothelial growth factor (VEGF) upregulation in wet AMD remains undefined [3,53,54]. There is evidence suggesting a role for both focal hypoxia and inflammatory cytokines. Ramrattan et al. described that older eyes, and especially those with AMD, exhibited significant loss of choriocapillaris, which would–at least theoretically–result in focal hypoxia [55]. This would be likely lead to regions of focal hypoxia in the overlying RPE. There is substantial evidence supporting the possibility of production of inflammatory cytokines as well as oxidative species in eyes with AMD [56,57]. While we use 1.0% oxygen as the hypoxic stimulus for the activation of our HREs, we found this promoter is sensitive to less severe hypoxia. Experiments are ongoing to characterize the response of this vector in conditions of less severe hypoxia (data not shown).

While hypoxia results in enhanced VEGF production, stimuli other than hypoxia may act via HIF-1α to induce VEGF and elicit neovascularization. Stress-induced molecules such as Interleukin 1-beta (IL-1β), tumor necrosis factor alpha (TNF-α), nitric oxide, lipid hydroperoxide, and transforming growth factor beta (TGF-β) all have been documented to signal through HIF-1 [58-62]. Oxidative stress induced by cobalt chloride or desferrioxamine has also been demonstrated to induce significant HIF-1α signaling [63,64]. Oxidative conditions also stimulate neovascular events [65,66]. Thus, our HIF-1-regulated vector would be activated by several proangiogenic conditions, including hypoxia, oxidative stress, as well as inflammation, all of which have been implicated in AMD. Our ongoing studies are examining the activation of the HRSE-6xHRE-Rpe65 promoter by oxidative stress.

The possible use of the HRSE-6xHRE-Rpe65 promoter as a platform for therapy against neovascularization is being tested in vivo in both AMD and proliferative diabetic retinopathy models. Both diseases are candidates for gene therapy because neovascularization is preceded by well defined markers of high risk. Although the construct we report is as strong as CMV, a major advantage of our strategy is that it is possible to vary the dosage of the transgene product by varying the number of HREs. Successful inhibition of CNV in murine models will further demonstrate the therapeutic potential of the HRE-driven endostatin [67] AAV. Experiments to test this promoter driving endostatin in a mouse model of CNV are in progress.

In summary, our data demonstrate hypoxia-induced AAV vectors for delivery specifically to the RPE. These vectors will be used to evaluate therapeutic efficacy of therapies targeted to these retinal locations. Moreover, because these vectors transform proangiogenic signal transduction pathways into switches for local delivery of antiangiogenic agents, they lay a foundation for new therapies that intervene in angiogenic diseases at their earliest stages, before tissue disruption or scar formation.
